# Low Alanine Aminotransferase Blood Activity, a Biomarker of Sarcopenia and Frailty, is Associated With Worse Post‐Total Laryngectomy Clinical Outcomes. A Retrospective Analysis of 427 Patients

**DOI:** 10.1111/coa.70047

**Published:** 2025-12-01

**Authors:** Yarden Tenenbaum Weiss, Keren Oren, Nofar Ben Mordechai Sharon, Tomer Kerman, Itai Hazan, Tal Marom, Oren Ziv, Gad Segal, Oded Cohen

**Affiliations:** ^1^ Department of Otolaryngology and Neck Surgery Samson Assuta University Hospital, Ashdod, Israel. Affiliated to the Faculty of Medicine, Ben‐Gurion University Beer Sheba Israel; ^2^ Ben Gurion University of the Negev Beer Sheva Israel; ^3^ Clinical Research Center Soroka University Medical Center and Faculty of Health Sciences, Ben‐Gurion University of the Negev Beer‐Sheva Israel; ^4^ Education Authority, Chaim Sheba Medical Center, Ramat‐Gan, Israel. Affiliated to the Faculty of Medicine Tel Aviv University Tel Aviv Israel

**Keywords:** alanine aminotransferase, frailty, head and neck cancer, prognosis, sarcopenia, total laryngectomy

## Abstract

**Introduction:**

Sarcopenia and frailty status have been shown to be associated with cancer patients' survival. Low alanine aminotransferase (ALT) blood activity was previously shown to be associated with frailty and poor clinical outcomes in cancer patients. This association was not addressed in head and neck cancer patients.

**Objectives:**

This was a retrospective study, investigating the possible association between pre‐operative low ALT blood levels and clinical outcomes in patients undergoing total laryngectomy due to malignancy.

**Patients and Methods:**

We did a retrospective analysis using the largest national Israeli HMO (Clalit health maintenance organization) database, including the demographic, clinical background, laboratory and clinical outcomes of head and neck cancer patients who underwent total laryngectomy.

**Results:**

A total of 427 patients were included. Patients were divided into two groups according to their ALT values: low ALT (0–10 IU/L, *N* = 100) and normal ALT (11–40 IU/L, *N* = 327). Low ALT levels were associated with significantly longer duration of in‐hospital stay (26 ± 17 vs. 23 ± 16 days, respectively; *p* < 0.001), higher probability of 1‐month, post‐discharge unplanned visits to the emergency department (RR = 1.66; *p* < 0.05) and worse 1‐year survival rate (67% vs. 77%, respectively; *p* < 0.05).

**Conclusions:**

Low, pre‐operative ALT levels are associated with worse prognosis for total laryngectomy patients. ALT levels may be used for better, personalised medicine by surgeons.

## Summary

Low, pre‐operative ALT blood levels are associated with worse prognosis for total laryngectomy patients. Blood test for ALT levels may be used for personalized preoperative assessment and prognostication.

## 1 Introduction

### 1.1 Predicting the Prognosis of Post Total Laryngectomy Cancer Patients

Total laryngectomy (TL) represents a major operation used in the realm of resectable head and neck malignancies. Survival of post‐TL patients depends both on operative results and the fate of the primary disease as manifested by the combined results of the operative technique and the pathology (staging) of the lymph nodes extracted during the accompanying neck dissection [1]. Ferrandino et al. have detailed several risk factors found to be associated with 30‐days readmission in TL patients: liver disease, valvular‐heart disease, co‐morbid coagulopathy and concurrent surgical procedures [2]. Disease grade and staging serve as tools in the realm of precision medicine. Nevertheless, simple, objective and reliable prognostication tools in personalised medicine are scarce.

### 1.2 Sarcopenia and Frailty as Predictors of Poor Prognosis in Cancer Patients

Survival of cancer patients, especially those with operable tumours, depends, among other variables, on their sarcopenia and frailty status. In their retrospective analysis of 84 TL patients, Salati et al. did not succeed in demonstrating an association between sarcopenia, as manifested by low muscle mass demonstrated in computed tomography, and clinical outcomes [3]. In contrast, Kitano et al. demonstrated the association between sarcopenia and poor clinical outcomes in patients with hypopharyngeal and laryngeal cancer going through TL. Their bottom line was that sarcopenic patients should be selected for less radical surgery [4]. Colback et al. succeeded in associating sarcopenia with poor prognosis of TL patients. However, they relied on low Striated Muscle Index (SMI) as demonstrated by computed tomography – rendering recurrent assessments less available [5].

### 1.3 Low ALT as a Biomarker for Sarcopenia and Frailty

In contrast to cases with elevated ALT blood activity levels, which are ascertained to indicate liver tissue damage (i.e., hepatitis), low‐normal levels of ALT activity in the blood have been shown to be associated with lower‐than‐normal total‐body muscle mass. Accordingly, low ALT blood levels were previously shown to be associated with frailty and poor clinical outcomes in a variety of scenarios: poor outcomes of cardiac and geriatric rehabilitation, shorter survival of patients with heart [6, 7] and lung diseases [8] and also poor prognosis of cancer patients, both hematologic [9] and solid tumours [10].

### 1.4 The Aim of the Current Study

In the current study, we aimed to demonstrate the association between pre‐operative sarcopenia and frailty, as manifested by low ALT blood levels and poor clinical post‐TL outcomes. Our aim was to better personalise the choice of surgical procedures for our future patients.

## 2 Methods

### 2.1 Patient Population

All patients included in this study suffered from head and neck cancer. All of them went through total laryngectomy surgery and were included after all relevant data needed for this study was available in their medical records. All medical records included in this study are routinely used for medical treatment purposes.

### 2.2 Descriptive and Analytical Statistics

Categorical variables were described as numbers and percentages. Continuous variables were divided according to their distribution (either normal or skewed, determined by QQ plotting). When normal variables were described using mean ± standard deviation, and when skewed, we used median and IQR (inter‐quartile range). For the purposes of univariate analysis calculation, differences in length of stay and number of post‐TL visits to the Emergency Department were calculated using Poisson regression, adjusted for the length of follow‐up with an offset term (log‐transformed follow‐up time). This accounted for variations in follow‐up durations and helped mitigate potential bias due to early mortality or incomplete follow‐up.

Statistical significance was determined when the *p*‐value was lower than 0.05. Statistical analysis was performed using IBM SPSS Statistics software.

## 3 Results

**FIGURE 1:**
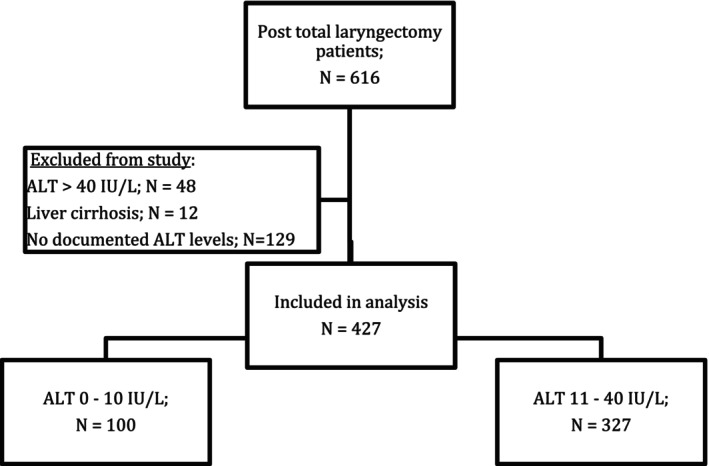
Study cohort CONSORT flowchart.

After approval by an Institutional Review Board (IRB approval #SOR‐0046‐24), patients' electronic medical records (EMRs) were accessed. Informed consent was waived by the IRB due to the retrospective nature of this study. All EMRs are used for routine clinical purposes. Figure 1 presents this study CONSORT patients' flow: Initially, data of a total of 616 patients who went through total laryngectomy procedures (ICD‐9 code 30.3 – complete laryngectomy) between the years 2000 and 2022 were extracted. We excluded the following patients: those with a known diagnosis of liver cirrhosis (assumed to have skewed ALT levels), patients with no documentation of blood ALT levels and patients with higher‐than‐normal ALT levels (> 40 IU/L), once again, assumed to be skewed by variable forms of hepatitis. All the above were excluded in order to stay with ALT levels that are associated with patients' striated muscular mass, reflecting the robustness/frailty status.

A total of 427 patients were included in our analysis, with 327 patients having ALT levels of 11–40 IU/L (normal) and 100 patients with ALT ≤ 10 IU/L (low—normal).

Table 1 presents our patients' cohort characteristics according to their pre‐operative ALT blood levels. Patients' age ranged between 32 and 95 years, with a similar median age of 70 in the lower ALT group and 68 in the normal ALT group. In both groups, the majority of patients were male Jews. The most common background diseases in both groups were arterial hypertension (49% in the low ALT group and 47% in the normal ALT group) and diabetes mellitus (31% in the low ALT group and 23% in the normal ALT group). Other background diseases we examined were chronic heart failure, dementia and chronic kidney disease. We also compared blood haemoglobin and creatinine levels between the two groups, as detailed below.

**TABLE 1:** Patients' baseline characteristics.

Characteristic	Lower ALT 0–10 IU/L, *N* = 100	Normal ALT 11–40 IU/L, *N* = 327	*p*
Patients' demographics			
Age median (IQR), years	70 (57, 78)	68 (60, 75)	0.6
Male sex, *n* (%)	82 (82)	282 (86)	0.3
Ethnicity, *n* (%)			0.7
Jew	84 (84)	280 (86)	
Arab	14 (14)	37 (11)	
Socioeconomic level, *n* (%)			0.2
Low	20/92 (22)	58/315 (18)	
Medium	68/92 (74)	224/315 (71)	
High	4/92 (4)	33/315 (11)	
Background diagnosis			
Dementia, *n* (%)	6 (6)	14 (4)	0.4
Hypertension, *n* (%)	49 (49)	154 (47)	0.7
CKD, *n* (%)	20 (20)	40 (12)	0.05
Chronic heart failure, *n* (%)	17 (17)	29 (9)	0.02
Diabetes mellitus, *n* (%)	31 (31)	76 (23)	0.12
Laboratory parameters			
ALT Median (IQR); IU/L	9 (7, 10)	17 (14, 23)	< 0.001
Haemoglobin median (IQR), (g/dL)	13.1 (11.58, 13.93)	13.1 (11.6, 14.37)	0.5
Creatinine median (IQR), (mg/dL)	0.9 (0.75, 1.12)	0.85 (0.7, 1.04)	0.2
Clinical outcomes			
Length of in‐hospital stay, Median (IQR) days	22 (15, 35)	19 (13, 30)	< 0.001
ED visits, *n* (%)			
1 month	22 (22)	55 (17)	0.044
3 months	37 (37)	107 (33)	0.057
Survival, n/N (%)			
1 year	67/100 (67)	253/327 (77)	0.036
2 years	48/97 (49)	188/313 (60)	0.065
5 years	23/82 (28)	108/276 (39)	0.067

Relating to the clinical outcomes data presented in Table 1, we made a univariate analysis of our cohort study population: In the lower (0–10 IU/L) ALT levels group, the length of in‐hospital stay was longer than in the higher (11–40 IU/L) ALT levels group (median 22 days vs. 19 days; *p* < 0.001). The number of post‐discharge Emergency Department visits was also lower in the lower ALT group during the first month (22 vs. 55; *p* = 0.044) and 3 months after the surgery (although without statistical significance: 37 vs. 107; *p* = 0.057). Survival rates were lower in the lower ALT group during the first year (67% vs. 77%; *p* = 0.036), 2 years (without statistical significance: 49% vs. 60%; *p* = 0.065) and 5 years after the surgery (without statistical significance: 28% vs. 39%; *p* = 0.067).

**FIGURE 2:**
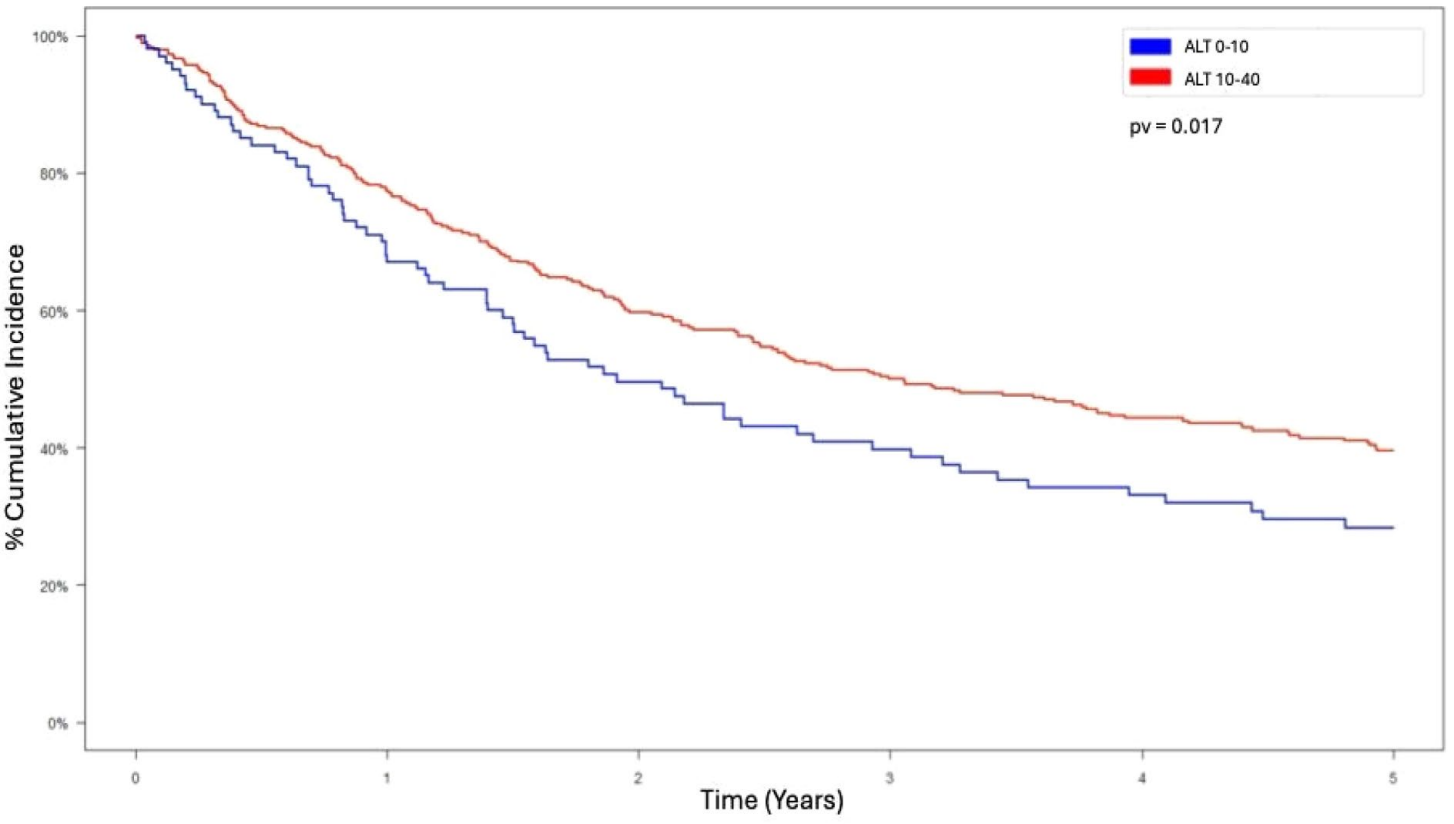
Kaplan‐Meier survival curve.

Cumulative survival was significantly worse in patients with low ALT levels than in patients with normal ALT levels, as shown in Figure 2.

We used a multivariable Cox proportional hazards regression in order to assess the association between ALT levels (0–10 IU/L vs. 11–40 IU/L) and mortality in post‐total laryngectomy patients, adjusted for age and gender, as shown in Table 2. The hazard ratio was increased in low ALT levels (HR = 1.35; *p* = 0.034). When adjusted, male sex was not associated with a statistically significant difference.

**TABLE 2:** Multivariable cox regression analysis of mortality by ALT levels, age, and sex in post‐total laryngectomy patients.

Patients' characteristic	HR	95% CI	*p*
ALT (0–10 vs. 11–40)	1.35	1.02–1.78	0.034
Age (each year)	1.02	1.01–1.03	< 0.001
Male sex	1.21	0.84–1.74	0.3

## 4 Discussion

Assimilating perspectives and tools of personalised medicine is an expanding, important trend in all clinical disciplines. Personalization of diagnosis, prognostication and treatment should go hand in hand with the ever‐expanding realm of precision medicine. While precision medicine concentrates on the disease/diseased tissues, personalised medicine puts the whole gestalt of patients at the centre of the physicians' interests.

Recent years have seen significant progress in the precision medicine tools and methodologies in the clinical management of patients who suffer from head and neck malignancies: For example, Shibata et al. [11] discussed 29 completed and ongoing clinical trials using personalised cancer immunotherapy for head and neck squamous cell carcinoma, from 1995 to the present day, showing remarkable progress in the field of ‘tailor‐made’ anti‐tumour therapy. FDA‐approved next‐generation genomic sequencing (NGS) platforms are increasingly used by oncologists treating head and neck squamous cell carcinoma patients, thanks to cost reduction trends and growing insurance coverage for NGS [12]. Personalised radiation therapy using advanced technologies such as stereotactic radiation and particle beam radiation allows for a more precise radiation dose, hence reduced radiation damage to surrounding tissues and the resulting side effects [13]. Automated planning of radiation therapy has been shown to deliver a reduced dose and less damage to surrounding tissues versus manually planned radiation for head and neck patients, while significantly reducing planning time [14].

Notwithstanding the above, personalised medicine should progress into the field of head and neck malignancies, with appreciation and assessment of frailty and sarcopenia as the front edge.

Low ALT blood levels were found to be associated with frailty and poor survival rates, independent of age, nutritional status, kidney function and significant comorbidities [8, 9, 10].

Several studies whose results were published in recent years have demonstrated the benefits of using ALT blood activity measurements as a tool for sarcopenia and frailty assessment in both benign and cancer patients: Segev et al. demonstrated the association between low ALT blood levels and shorter survival of patients with heart diseases [6, 7], while Lasman et al. showed low ALT values associated with poor, long‐term survival and increased risk of mortality among hospitalised patients due to COPD exacerbation [8]. Several authors reported low ALT blood levels as a predictor of survival in cancer patients: in a retrospective cohort study of 716 patients, Hellou et al. showed that blood ALT measurements could potentially point out differences in CLL patients' prognoses. Laufer et al. reported similar results in a retrospective data analysis of 3075 patients demonstrating an association between low ALT blood levels and decreased survival of bladder cancer patients [9].

In the current study, we demonstrated the fact that low ALT levels succeeded in differentiating patients that had different clinical outcomes. Low, pre‐operative ALT levels, indicative of sarcopenia and frailty, were associated with significantly longer duration of in‐hospital stay, higher probability of 1 month, post‐discharge unplanned visits to the emergency department and lower 1‐year survival rate. The above results demonstrate the feasibility of using ALT measurements in the task of prognostication of patients who are candidates for TL.

## 5 Conclusions

Baseline, low ALT blood activity levels are independently associated with the clinical outcomes of head and neck malignancy patients and could serve as a means for better patients' preparation and treatment planning. It is recommended that surgeons taking care of such patients assimilate this simple and accessible tool as part of their pre‐operative evaluation, serving as an embodiment of personalised medicine in their clinical practice.

## 6 Limitations

This was a retrospective study without comparative interventions. Further research should question the different treatment modalities that should be applied in either low‐ or normal ALT level patients. Also, we excluded patients with higher‐than‐normal ALT levels, and this is a population of patients for whom the results of our study should not be applied.

## Author Contributions

Y.T.W., K.O., O.C., O.Z. and G.S. conceptualised the idea for this study, analysed the data, wrote the manuscript, critically reviewed the manuscript and approved the final manuscript as submitted; T.K. and I.H. analysed the data and critically reviewed the manuscript; N.B.M.S. and T.M. critically reviewed the manuscript and approved the final manuscript as submitted. All of the authors provided critical feedback and helped shape the research and manuscript.

## Ethics Statement

The study was approved by the Institutional Review Board (IRB approval #SOR‐0046‐24).

## Conflicts of Interest

Tal Marom and Oded Cohen are members of the Clincal Otolaryngology international editorial board.

## Data Availability

The data that support the findings of this study are available on request from the corresponding author. The data are not publicly available due to privacy or ethical restrictions.
